# Progress in Surgery

**Published:** 1897-10-23

**Authors:** 


### Progress in Surgery.

### HERNIA.

(*Continued from page* 28.)

**Femoral Hernia.**—Dr. Leonard Molloy (Blackpool) calls attention to the enlargement in femoral herniæ of that lymphatic gland which normally occupies the crural ring,[26] and he gives three cases as examples where there was a difficulty in diagnosis as to whether the tumour was gut or a solid growth. Dr. Edebohls read a paper describing the surgical operation for femoral hernia, and advocates the suture of Poupart's ligament to the periosteum of the horizontal ramus of the pubes. The irritation of the periosteum produced tends to new formation in and about the crural ring, which will constitute an efficient barrier against the recrudescence of a hernia. The saphenous opening may then be closed by sewing the falciform process to the pubic portion of the fascia lata. The operation is done through the posterior wall of the inguinal canal, which is afterwards closed by Bassini's method. The operation is indicated when a femoral hernia and an inguinal hernia coexist.[27] Dr. Stinson's operation for inguinal hernia has already been referred to. He also reports five cases of the radical cure of femoral hernia.[28] Anatomically strong fibres reinforce the internal ring—the inner and outer limbs of transversalis fascia—and Stinson urges that the entire opening should be closed and the peritoneal sac should be previously cut off and peritoneum sutured, not left as a stump like a wedge in the internal ring. He holds that it is as important to close the femoral ring and canal as the internal inguinal ring and canal. The falciform and pectineal fasciæ should not be sewn together, as it is unnecessary as well as dangerous, he thinks. The same objections apply to osteoplastic operations. The operation he recommends is to make an incision parallel with Poupart's ligament and half an inch below it; the femoral canal should then be cleared of all fat, glands, &c., the peritoneum stitched after cutting the sac away, and the canal closed by suturing together the anterior and posterior layers of the femoral sheath at the femoral ring, including also some of Gimbernat's ligament. The saphenous opening is then stitched up.

Mr. Stanmore Bishop had a case of strangulated femoral hernia without external signs—only detected by cœliotomy.[29] Mr. Bidwell reported two cases of irreducible femoral hernia with the vermiform appendix in the sac.[30] Mr. William Rose has recorded a case of strangulated femoral epiplocele which underwent spontaneous cure by sloughing of the tissues, and two cases in which the vermiform appendix was found in the sac of a femoral hernia.[31] In a case of gangrenous strangulated femoral hernia Mr. Makins relieved the stricture by cutting from without inwards through Poupart's ligament.[32]

**Umbilical Hernia.**—This subject has been fully dealt with by Sebileau.[33] He gives the following classification:—

1. Hernia neonatorum.—This includes (*a*) complete prolapse of the viscera; (*b*) partial ventral hernia, in which the umbilical vessels and cord will require ligature; (*c*) small umbilical hernia.

2. Hernia in children.—This is classed according to the ages of the children: (*a*) crying period; (*b*) walking period; (*c*) older children.

3. Hernia in adults.—(*a*) When operation is compulsory owing to strangulation; (*b*) when no complication is present. In early life operation is not necessary, but after seven years of age it becomes imperative, and all umbilical herniæ in adults seem to have originated in childhood. For severe cases the author performs omphalectomy. An ingenious operation for a very large ventral hernia has been described by Dr. George Noble, Atlanta.[34]

**Obturator Hernia.**—Dr. Monro Grier, Cornwall, recorded a case of obturator hernia successfully reduced by copious enemata, one enema of "a gallon" having been given.[35] Dr. Sinigar reported a case of strangulated obturator (Richter's) hernia at the Marylebone Infirmary,[36] the accurate diagnosis only proving possible after death. An interesting series of three cases of strangulated obturator hernia was given by Mr. Godlee.[37] He mentions that the right ovary and Fallopian tube have been recorded as present in obturator herniæ. All three cases were in elderly women, were partial in character, and were fatal in termination. Mr. Godlee, from his experience, is in favour of making an abdominal section, as well as operating through Scarpa's triangle, in order to determine the condition of the gut, which is so often gangrenous.

**Strangulated Cæcal Herniæ.**—Cases of this uncommon condition are recorded by Mr. Mayo Collier at the North-West London Hospital,[38] who returned the gut to the abdomen and performed a radical cure by Halsted's operation; and by Dr. Walker (New York), who found the cæcum and appendix in a strangulated femoral hernia.[39]

**Rare Forms of Hernia.**—An ischio-rectal hernia, due to dislocation of a subperitoneal fibroma, is recorded by Mr. Lynn Thomas. He removed the fibroma, which weighed over two pounds.[40] Herniæ of the bladder have been recorded by Mr. Thelwall Thomas[41] and by Dr. Gibson, of New York.[42] The latter has seen four cases in a year, and gives a table of cases, adding 45 cases to the 58 already recorded by Curtis. The total mortality appears to be about 30 per cent., and the real mortality connected with the operation itself about 12 per cent. The rare condition of strangulated right ovarian hernia was met with by Mr. John Morgan,[43] and the very rare one of hernia of the Fallopian tube by Mr. Thelwall Thomas.[44] In the latter the ovary was not seen at all.

Tuberculous herniæ are usually inguinal, and may be primary or secondary (Renault[44]); they are usually herniæ of old standing which have been subject to traumatism. E. Roth (Heidelberg) has published three such cases.[45] Recovery from the tuberculous condition is not uncommon.

Muscular hernia due to rupture of the aponeurosis has been referred to by Janni, who thinks, however, that the contraction of a muscle may not be the usual cause of rupturing its sheath. In a patient who had recurrent pain from rupture of the sheath, as proved at the operation, there was at no time any hernia of the muscle.

[26] Lancet, Feb. 27, 1897. [27] Med. Record, Jan. 23, 1897. [28] N. Y. Medical Record, Dec. 1896. [29] Clinical Journal, Nov. 4, 1896. [30] Medical Week, May 28, 1897. [31] Lancet, June 20, 1896. [32] Lancet, Dec. 19, 1896. [33] Sem. Méd., Dec., 1896; and Jan., 1897. [34] Amer. Med. Surg. Bulletin, June 20, 1896. [35] Lancet, June 27, 1896. [36] B.M.J., Mar. 6, 1897. [37] Lancet, June 26, 1897. [38] Lancet, Dec. 5, 1897. [39] N. Y. Med. Record, Feb. 13, 1897. [40] Lancet, July 24, 1897. [41] B.M.J., Oct. 24, 1896. [42] Med. Record, Mar. 20, 1897. [43] Lancet, May 15, 1897. [44] Jour. de Méd., Mar. 25, 1869. [45] "Annals of Surgery, March, 1897. [46] Rif. Med., Nov., 1896.

### GENITO-URINARY SURGERY.

(*Continued from page* 46.)

**Catheterisation of Ureters.**—This method of diagnosis is fully established by means of Kelly's instruments in the case of the female. In the male much greater difficulty is experienced in every way, but yet much promise of future success is foreshadowed. The work of Dr. Willy Meyer has borne much fruit, but at the same time the elaborate and fanciful diagnoses said to be made by ureteral catheterisation may be taken *cum grano*. For instance, here are some diagnoses: "Possibly slight irritation of the renal pelvis;" "catarrh of the renal pelvis, probably a very moderate pyelitis and the cause of a very moderate renal hyperæmia, moderate chronic cystitis, excessive crystalline deposit, allowing suspicion of renal stone."[14]

Kelly's method of ballooning the bladder with air and inspecting its interior through straight tubes passed through the urethra is for ever excluded in the male, in whom cystoscopy and catheterisation of the ureters can only be done under the principles of Nitze's instrument. For this three conditions are essential: (1) The urethral calibre must be sufficient; (2) the bladder must have a capacity of at least four ounces; (3) the fluid in the bladder must be transparent and remain so. The ureter cystoscope used by Dr. Meyer is Casper's, and it is strongly recommended by him. The process is not without dangers of its own probably, apart from the slight risks of manipulations within the bladder. In one of Dr. Meyer's cases he failed to draw off one drop of urine, and learned to his disgust that the tip of the catheter had been bent a little at the eye, and that within it a small shred of coagulated blood had been caught. This is described as a "perfectly successful catheterism of both ureters," but most surgeons would regard such signs as those of a false passage of the ureter—a condition serious enough in all conscience. Even with skill some bleeding usually occurs. It is interesting to note that Dr. Meyer has observed the unilateral excessive production of uric acid, *i.e.*. confined to one kidney only. The manipulation is said to be easy, but requires experience and practice, and takes time, even as long as two hours. In order to obtain a sufficient quantity of urine it is advisable to make the patient drink freely beforehand. The following rules are recommended: (1) Wash and cocainise the bladder. (2) Inject about six ounces of clear fluid. (3) Introduce the instrument, afterwards fully closing the lid. (4) Approach a ureteral opening, letting it appear at the very end of the cystoscopic picture. (5) Push the catheter forward in a direction parallel with the ureter. (6) Having passed it for one to two inches, withdraw the wire mandrel and collect the urine from the ureter. These observations refer, of course, to the male.

The quantities of urine obtainable are very small, therefore special methods of analysis are requisite, and Dr. F. E. Soudern, who has analysed Dr. Willy Meyer's specimens for him, has given us the benefit of his methods, based upon the assumption that in a given case six cubic centimetres of urine can be obtained from each ureter. He uses two sterilised glass tubes labelled "right" and "left," with space for writing other particulars. The six cubic centimetres are poured into the receptacle of a Westphal's specific gravity balance, then the sediment of the whole is obtained by a centrifugal machine for routine microscopic examination and staining with micro-organisms. The urine is then filtered and reaction obtained. One cubic centimetre is used for the test for albumin, and one for Esbach's test. If albumen be present the remaining three cubic centimetres are boiled, and the albumen filtered off. One cubic centimetre is used for the estimation of urea, half a cubic centimetre (diluted) for the sugar test (by Whitney reagent preferably). The remainder serves for estimation of chlorides or any special constituent. In addition the quantity, colour, and odour of the urine must be estimated.

**Electric Cystoscopy.**—The value of the cystcscope in lowering the mortality of nephrectomy is dwelt upon by Mr. Hurry Fenwick with an experience of a hundred cases.[15] No patient with tuberculous deposits in epididymis or prostate or malignant disease of the bladder should be cystoscoped. In women Kelly's method of direct observation through specula is probably more certain. Cystoscopy is indicated in cases of renal hæmaturia or of severe symptomless pyelitis, and may be performed with advantage in the patient's own urine if it be sufficiently clear.

**Vesical Calculus.**—Dr. Ramon Guiteras has given an estimate of the comparative mortality of operation taken from nearly 3,000 cases.[16]

| | Perineal Lithotomy. | Suprapubic Lithotomy. | Litholapaxy. |
|---|---|---|---|
| Under 14 | 2¾ | 12½ | 1½ |
| 14 to 50 | 11½ | 12 | 3½ |
| Over 50 | 16½ | 32 | 6 |

**Cases and Observations.**—Drs. William White and Alfred Wood, of Philadelphia, read a paper before the College of Physicians of Philadelphia[17] upon the diagnosis and treatment of surgical affections of the kidneys with illustrative cases. Symptoms of renal calculus may exist from other causes, as in the case of a woman who had both ovaries removed for what turned out to be a renal calculus. Spinal caries is very prone to give symptoms simulating renal calculus. Again, renal calculus may be present without any symptoms, and may even be undetected by palpation with the kidney between the fingers. Abscess of the kidney may resemble a stone. Early operation is pleaded. "It has long been recognised that the mortality following nephrotomy is much less than that after nephrectomy. If, therefore, the disease be attacked at a time when the former operation is still appropriate, the patient's chances are very much better than if operation is delayed." Nephrotomy is urged for anuria, seeing that it reduces the mortality from 71 to 37 per cent. Seventeen cases are given in full detail.

The results of 66 renal operations are given by Albaran.[18] He observed, after a number of his operations, reflex phenomena of a very grave nature, always accompanied by an oliguria of more or less marked character. The symptoms were incoercible vomiting, epigastric pain, pallor, small pupils, small rapid pulse. They occurred within the first 24 hours, and resisted all forms of treatment. Death may occur without obvious post-mortem signs accountable for it. This fully accords with the opinions of those who have had large experience in renal surgery.

A contribution to the surgery of the kidney and ureter has been given by Dr. Serster, of New York. In a case of hydronephrosis due to a contracted renal orifice, he performed a plastic operation, opening out the orifice like a funnel by radial incision and plastic suture, thus curing the hydronephrosis without nephrectomy. Extirpation of the sac of a hydatid is condemned, owing to the intimate connections with organs in the vicinity. He urges the earlier performance of nephrotomy in acute inflammatory conditions of the kidney, such as acute albuminuria with swelling of the kidney. Early operation relieves tension, and also suppuration if present, and is a comparatively safe procedure. The application of surgery to acute nephritis is a new departure with a prospect of salvation in store for it.

[14] N. Y. Med. Record, May 1, 1897. [15] B. M. J., Feb. 27, 1897. [16] N. Y. Med. Journal, March 20, 1897. [17] Annals of Surg., Jan., 1897. [18] Rev. de Chir., Nov. 10, 1896.

---

### LIVER, GALL-BLADDER, AND DUCTS.

The surgery of the liver and gall-bladder has made rapid strides. The method of preliminary ligature to liver substance before removal of growths has given an impetus by reducing the tendency to that alarming hæmorrhage which is the great obstacle to hepatic surgery. The study of the gall-bladder, its pathological relationships and possibilities, coincident with the introduction of various mechanical means and methods, has opened new channels for successful surgery.

### Liver.

**Prolapse.**—A case of anteversion of the liver upon its transverse axis is recorded by Ferrari[1] in a woman aged 24, causing much pain in the erect attitude and emaciation. The liver was kept in its abnormal position by strong adhesions. These were divided, and the liver thus set free was fixed in its normal position by four catgut sutures passed through the entire thickness of the liver substance. The liver was moreover supported awhile by iodoform gauze packing beneath its under surface, and the pelvis was kept highly raised for a month. Six months afterwards the patient was free from pain.

**Rupture.**—Dr. Vanverts has written a paper upon this subject.[2] He describes four stages after an abdominal injury when severe: 1st, the preliminary collapse, not always present; 2nd, stage of uncertainty as to diagnosis; 3rd, signs of increasing anæmia from hæmorrhage; 4th, complications. The collapse should be treated in the usual way, the patient kept at absolute rest, constantly and carefully watched, and cold applied locally to the liver. If signs of internal hæmorrhage appear the abdomen should be opened by a large incision, and the liver explored. Any rupture is best treated by gauze plugging left in for two to four days. Severe hæmorrhage is best arrested by sponge pressure. If any large vessel be seen it can be ligatured. Deep sutures are useful in some cases. When peritonitis is present a remote chance for the patient may be given by opening and draining the abdominal cavity.

**Abscess.**—This disease is common enough in the tropics, but rare in Great Britain. Dr. James Gemmel, of the County Asylum, Lancaster, records a case with "aspiration; recovery"[3]; but as, after aspiration, the abscess re-collected and burst into the colon ending in recovery, the case is no argument in favour of that objectionable mode of treatment. Seven consecutive cases treated by incision have been detailed by Walder

Boyd, of the Colony Hospital, Grenada, W.I.[4] The majority occurred in the right lobe, with adhesions, and the biliary function was not much interfered with. The incisions were free, permitting digital exploration, and rigorous antiseptic precautions were always observed. The cavities were flushed out with sterilised water and packed with iodoform gauze. The average stay in hospital was one month, and in only one of the cases was the prognosis doubtful.

**Hydatid Cysts.**—Dr. A. Chauffard[5] recorded a case of sudden death within a few minutes of "simple" exploratory puncture of a hydatid cyst, the patient being a vigorous man. Soon after the exploration with an ordinary Pravaz syringe he felt suddenly ill, turned and tossed from side to side as if in anguish, and began to scratch himself in various places. An epileptiform seizure then took place followed by others, and death appeared to occur from exhaustion and asphyxia. Post-mortem: There was marked compensatory hyperplasia of the left lobe of the liver, the hyperplasia originating microscopically in the portal-biliary spaces. Clinically, the termination was an aggravated form of what occasionally occurs when a hydatid cyst ruptures, or is tapped. "L'intoxication hydatique" (Achard), "L'urticaire hydatique" (Debove) appears to be an acute toxic poisoning, the nature of which is quite unknown, and experimental investigations have hitherto failed to assist in an elucidation. Moral—do not aspirate hydatid cysts of the liver.

Many cases of successful drainage of hydatid cysts by free incision are never recorded. A case is noted by Mr. J. Small,[6] of Geelong, Victoria, in which, owing to profuse hæmorrhage at the first attempted operation treatment had to be deferred; the second operation was done by opening the liver with Paquelin's cautery. Again there was serious hæmorrhage, but eventually all difficulties were overcome, and the patient recovered. Mr. Betham Robinson[7] described a case of hydatid of the liver involving the right pleura and peritoneal cavity. The patient was first of all tapped to draw off the ascitic fluid, which proved to contain hooklets. When the abdomen was opened the hand could be passed through a hole in the diaphragm into the right pleura, reaching the dome above. The patient recovered.

**Partial Resection of the Liver.**—This subject has been studied clinically and experimentally by Kousnetzoff and Pensky.[8] They recommend that the liver substance be tied pretty tightly, as it is elastic, and that it should be returned into the abdomen, not treated extra-peritoneally. If blunt needles be used and the ligatures be tied slowly but tightly hæmorrhage may be completely arrested. Omental grafts will not always prevent secondary hæmorrhage. The best incision for giving free access to the liver is one parallel to the false ribs.

David Greig, of Dundee,[9] reports a case of empyema of the gall-bladder with stone in the cystic duct treated by cholecystectomy, and partial resection of the liver. In this case ligature of the cut liver inch by inch proved effectual in the control of hæmorrhage. It is not always a simple matter to prevent hæmorrhage from the liver. Malignant disease may be very vascular, and there are tumours purely vascular. Grave hæmorrhage may follow puncture with a trocar, as in a case recorded by Dr. Ricard,[10] in which he was obliged to resort to the actual cautery. The tumour was probably a vascular sarcoma.

[1] Supplem. al Policlinico, Dec. 26, 1896. [2] Arch. Gén. de Méd., Jan., 1897, p. 44. [3] Lancet, Dec. 19, 1897. [4] Brit. Med. Journ., Aug. 21, 1897. [5] Medical Week, Aug. 7, 1896. [6] Intercolon. Med. Journ. of Australasia, July 20, 1896. [7] Lancet, Oct. 17, 1896. [8] Rev. de Chir., Dec., 1896. [9] Scottish Med. and Surgical Journal, Jan., 1897. [10] Medical Week, Jan. 22, 1897.

